# Recent trends in basil seed gum-based films and coatings for food packaging: An updated review

**DOI:** 10.1016/j.fochx.2025.102937

**Published:** 2025-08-21

**Authors:** Shahriyar Sahraeian, Hadi Hashemi, Fatemeh Ghiasi, Mohammad-Taghi Golmakani, Shahriyar Valizadeh, Reza Tahergorabi, Yana Artemovna Firsukova, Amin Mousavi Khaneghah

**Affiliations:** a Department of Food Science & Technology, Agricultural College, Shiraz University, Iran; b Food and Nutritional Sciences Program, North Carolina Agricultural and Technical State University, Greensboro, NC, USA; c Faculty of Biotechnologies (BioTech), ITMO University 191002, 9 Lomonosova Street, Saint Petersburg, Russia; d Department of Food Science and Nutrition, Faculty of Food Engineering, University of Campinas, Campinas, SP, Brazil

**Keywords:** Basil seed gum, Coatings, Edible films, Food industry, Shelf life, Sustainability

## Abstract

Basil seed gum (BSG) is a natural, biodegradable hydrocolloid derived from basil seeds with promising applications in food packaging. Due to its biocompatibility, film-forming capacity, and favorable mechanical and barrier properties, BSG is an eco-friendly alternative to synthetic packaging. This review highlights the development and functional properties of BSG-based films and coatings, especially for preserving perishable foods like seafood, meat, poultry, fruits, and fried products. BSG films offer excellent protection against moisture, oxygen, and microbial growth, enhancing food safety and shelf life. They also improve the sensory attributes of food, including texture and appearance. Incorporating bioactive compounds can add antimicrobial and antioxidant benefits. These sustainable films support waste reduction and offer cost-effective packaging solutions. However, further studies are needed to refine formulations and validate large-scale applications. Collaborative efforts between researchers and industry stakeholders are essential to advance BSG's commercial use in sustainable food packaging.

## Highlights

• BSG has hydrophilicity, biodegradability, and versatile functionalities for coatings and films.
• BSG, being natural, is safe, non-allergenic, and offers potential health benefits.
• Films and coatings made from BSG effectively extend the shelf life of food.
• Modifying BSG (such as esterification) can improve the properties of the coating.

## 1 Introduction

Gum is a natural exudate from certain plants and trees, primarily composed of polysaccharides and biopolymers. The extraction process involves tapping or slashing the plant's bark, allowing the gum to ooze out, which is then collected and processed. Gum extraction from plant seeds consists of the hydration of seeds, followed by filter pressing to separate mucilage from the seeds. The significance of gums lies in their versatile applications, particularly in the food industry, where they serve as thickening, stabilizing, and gelling agents (Gahruie et al., 2021). It is important to differentiate between the gum fraction of basil seeds and general aqueous or ethanolic extracts. While extracts may contain phenolic compounds and other bioactives, they lack the high-molecular-weight polysaccharides that provide the essential rheological and film-forming properties of basil seed gum. The purified gum, obtained through hydration and separation of mucilage, offers specific techno-functional attributes such as viscosity control, mechanical integrity, and moisture/oxygen barrier properties that are critical in packaging applications.

The genus *Ocimum* (family *Lamiaceae*) comprises a diverse group of aromatic plants with broad geographic distribution and significant variability in both genetic and chemical composition. This genus includes between 35 and over 150 species, depending on classification criteria, and encompasses annual and perennial herbs and shrubs native to tropical and subtropical regions of Asia, Africa, and the Americas (Shiam et al., 2025). Among them, *Ocimum basilicum* L. (sweet basil) is considered the most widely cultivated and economically important species. Other commonly grown species include *O. gratissimum*, *O. tenuiflorum* (*O. sanctum*), *O. americanum*, and *O. africanum* (Yadav et al., 2025). These species vary in essential oil profiles, polyphenol content, and polysaccharide composition. Since the seed gum is a mucilage derived from the outer coat of the seed, its composition and functional properties—such as viscosity, solubility, and film-forming ability—can also vary across species (Shiam et al., 2025). Most of the available literature on basil seed gum-based films and coatings focuses on *O. basilicum*, with limited comparative data on other *Ocimum* species. Therefore, further research is warranted to investigate how interspecies variability may influence the performance of basil seed gum in food packaging applications (Hashemi Gahruie et al., 2017; Romano et al., 2022). Basil seeds themselves are nutrient-rich and contain significant amounts of carbohydrates (approximately 40.1 g/100 g), proteins (11.4–22.5 g/100 g), and lipids (16.6 g/100 g), along with dietary fiber (26.2 g/100 g) and ash (8.9 g/100 g). They are also abundant in bioactive compounds, including polyphenols, flavonoids, and essential fatty acids—particularly α-linolenic acid—contributing to their antioxidant and health-promoting properties (Calderón Bravo et al., 2021). The seed coat is the primary source of the mucilaginous polysaccharide (basil seed gum), which accounts for about 17–20 % of the seed weight (Calderón Bravo et al., 2021). The chemical composition of basil seeds can vary depending on species, cultivar, and growing conditions, which may in turn influence the yield and properties of the extracted gum (Guan et al., 2024).

**Fig. 1:**
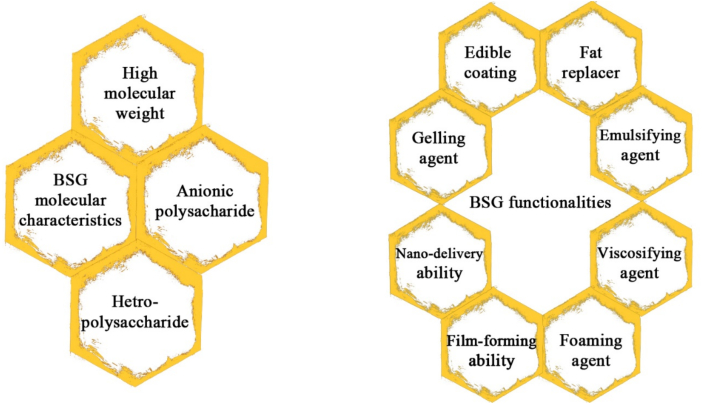
Functionalities and molecular characteristics of basil seed gum.

Due to their high dietary fiber content, basil seeds are commonly used in traditional drinks and refreshments for their curative properties. When exposed to an aqueous medium, they develop a mucilage layer that can be extracted and utilized as a natural gum. Basil seed gum (BSG) possesses distinct merits, including hydrophilicity, biodegradability, biocompatibility, and favorable rheological properties, making it an intriguing functional ingredient and a desirable choice for creating edible coatings and films (Hashemi Gahruie et al., 2017; Shooli et al., 2024). Based on their molecular weight, basil seed gum can be divided into two distinct fractions by gradual ethanol precipitation: precipitate (PER-BSG) and supernatant (SUPER-BSG). Basil seed gum is an acidic polysaccharide and contains approximately 6.51 % uronic acid molecules (comprising D-mannuronic and D-galacturonic acids). PER-basil seed gum, constituting approximately 69 % of the total basil seed gum, has a molecular weight of 5980 kDa and a uronic acid content of 3.84 %. SUPER-BSG has a molecular weight of 1045 kDa and a higher uronic acid content (13.39 %), resulting in a greater anionic character. Basil seed gum comprises L-arabinose, d-glucose, D-mannose, D-galactose, D-xylose, and L-rhamnose in relative proportions of 15, 25, 10, 15, and 5, respectively. Fig. 1 summarizes basil seed gum's functionalities and molecular characteristics (Naji-Tabasi & Razavi, 2017).

Numerous studies have demonstrated the diverse techno-functional potentials of basil seed gum, including emulsification capacity, foam-forming ability, gelling properties, and film-forming and coating abilities (Hashemi Gahruie et al., 2017; Hosseini-Parvar et al., 2016; Rafe & Razavi, 2013). Given the growing public awareness of the global environmental impacts of synthetic and non-degradable packaging materials, scientists are increasingly exploring natural biopolymers for packaging and coating. Ideal candidates for this purpose are biodegradable, biocompatible, and chemically neutral polymers that remain inert. Polysaccharides and proteins are frequently used as biodegradable packaging and coating materials (Aziz et al., 2024; Hashemi Gahruie et al., 2020). As natural biopolymers satisfy these criteria, researchers have been investigating the potential of various gums, such as basil seed gum, to serve as a natural and biodegradable food packaging and coating material source. Different food products, including meat, poultry, fried products, seafood, and fresh produce, have been studied to assess basil seed gum's capability as a packaging and coating material. The rationale for using basil seed gum as a material for films and coatings lies in its exceptional functional properties and compatibility with other biopolymers (Mortazavi Moghadam et al., 2023). Unlike synthetic polymers, basil seed gum is derived from a natural source, making it biodegradable and safe for human consumption (Oraç et al., 2023). Its ability to form flexible, transparent films with good mechanical and barrier properties positions it as a viable candidate for food packaging applications (Etemadi et al., 2020). Additionally, basil seed gum can be modified or blended with other polymers and additives to enhance its performance, such as improving water resistance or incorporating antimicrobial agents (Ansarian et al., 2022a; Hashemi Gahruie et al., 2019a). These characteristics make basil seed gum a valuable material for addressing challenges in food preservation, shelf-life extension, and sustainable packaging. Although edible films and coatings from various plant-based gums have been reviewed, there is no comprehensive and updated review study dedicated to basil seed gum-based films and coatings, despite the significant growth in publications over the past years. Existing literature reviews either briefly mention BSG in the context of general plant gums or focus mainly on its extraction and structural characterization without fully exploring its food packaging functionalities. This review addresses explicitly that gap by compiling and critically analyzing recent research on BSG-based films and coatings. This targeted approach aims to guide both researchers and industry stakeholders toward advancing the commercial use of BSG in sustainable packaging. Since there has been a noticeable increase in scientific interest in basil seed gum as a biopolymer for food packaging applications in recent years Table 1 presents the number of research articles published per year on BSG-based films and coatings by 2025, based on a keyword search (“basil seed gum” AND “film” OR “coating”) in the Scopus database to illustrate the growing trend. As shown, the number of publications has grown significantly since 2014, highlighting the emerging relevance of this biopolymer in sustainable packaging research.

**Table 1:** Annual number of scientific publications related to basil seed gum-based films and coatings by 2025. Data retrieved from the Scopus database using the keywords “Basil seed gum” AND (“Film” OR “Coating”) in titles, abstracts, and keywords (search conducted in July 2025).

Year	Number of publications	Title	Reference
2025 (by July)	11	Intelligent pectin and alginate-based biopolymeric film enriched with purple basil anthocyanins for pH-sensitive detection of chicken meat freshness	Adımcılar et al. (2025)
		Investigating the effects of basil (*Ocimum basilicum* L.) mucilage and cellulose nanofiber-based edible films with post-probiotics on fish fillet shelf life	Mokhtaran et al. (2025)
		Incorporation of co-encapsulated extracts of saffron petal and *Stachys schtschegleevii* into chitosan/basil seed gum/graphene oxide bionanocomposite: Effects on physical, mechanical, antioxidant, and antibacterial properties	Rajabi and Razavi (2025)
		Sustainable absorbent pads from polybutylene adipate terephthalate/thermoplastic starch films combined with hairy basil (*Ocimum basilicum*) powder to enhance meat shelf life	Khunta et al. (2025)
		Innovative active packaging with basil seed gum and probiotic *Lactiplantibacillus pentosus* v390 for strawberry preservation: preparation, characterization, and modeling	Rahmati-Joneidabad, Alizadeh Behbahani, Taki, Hesarinejad, and Toker (2025)
		Active packaging coating based on *Ocimum basilicum* seed mucilage and probiotic *Levilactobacillus brevis* Lb13H: preparation, characterization, application and modeling the preservation of fresh strawberries fruit	
		The Influence of Basil and Cinnamon Essential Oils on Bioactive Sponge Composites of Collagen Reinforced with Hydroxyapatite	Robu et al. (2025)
		Effect of curcumin loaded in nanoemulsion basil seed gum coating on the reduction of acrylamide and oil absorption in fried potato strips	Abdolshah et al. (2025)
		Alternative polysaccharide wound dressing from heat-moisture starch/basil seed (*O. basilicum* L.) mucilage with incorporated glycolic acid antimicrobial agent	Phonrat et al. (2025)
		Fabrication of electrospun polycaprolactone nanofibrous mats loaded with purple basil extract (*Ocimum basilicum* L.) as colorimetric pH indicator films	Erez et al. (2025)
		Preparation, characterization, and biological evaluation of *Ocimum basilicum* hemicellulose-based composites for wound healing	Waqar et al. (2025)
2024	13	Active microcapsules and edible films obtained from basil seed gum and ginger essential oil: Fabrication and characterization	Shaahbaz and Jouki (2024)
		Generation and evaluation of novel active biodegradable films based on modified basil seed gum and vitamin E nanoliposomes	Salehi et al., (2024)
		Improving oxidative stability of flaxseed oil by zein-basil seed gum electrospun nanofibers activated by thyme essential oil loaded nanostructured lipid carriers	Danaei Oskouei et al. (2024)
		Active packaging coating based on *Ocimum basilicum* seed mucilage and *Hypericum perforatum* extract: Preparation, characterization, application and modeling the preservation of ostrich meat	Zamani Faradonbeh et al. (2024)
		Effect of basil seed gum coating and ultrasound pretreatment on frying time, oil uptake, hardness, color indexes, and sensory properties of potato slices	F. Salehi et al. (2024)
		Development and investigation of a polysaccharide ternary nanocomposite based on basil seed gum/graphene oxide/anthocyanin for intelligent food packaging	Kafashan and Babaei (2024)
		Sustainable innovations in food packaging: Antioxidant basil-enriched cassava starch films with UV protection and enhanced water and mechanical resistance	Lacovone et al. (2024)
		Development of eco-friendly and edible adhesive using basil seed mucilage	Lee et al. (2024)
		Effect of carboxymethyl cellulose and montmorillonite addition on the physicochemical and thermal properties of basil seed mucilage-based biodegradable film	Qomi-Marzdashti and Zahedi (2024)
		Effect of vacuum packaging combined with edible basil seed gum coating containing lemon essential oil on shelf life extension of refrigerated shrimp (*Penaeus indicus*)	Shafiei et al. (2024)
		Producing polycaprolactone and basil seed gum nanofibers using an electrospinning process	Zamani et al. (2024)
		Basil seed mucilage as a bioadhesive polymer: Development of naproxen sodium microspheres and suppositories with in-vitro and ex-vivo studies	Tripathi et al. (2024)
		Natural wound dressing from acid-modified basil seed mucilage containing *aloe vera* extract	Seeharaj et al. (2024)
2023	10	Effects of basil seed and guar gums coatings on sensory attributes and quality of dehydrated orange slices using osmotic-ultrasound method	Eftekhari et al. (2023)
		Development of basil seed mucilage (a heteropolysaccharide) – Polyvinyl alcohol biopolymers incorporating zinc oxide nanoparticles	Hasheminya et al. (2023)
		Biodegradable edible film based on basil seed gum: The effect of gum and plasticizer concentrations	Oraç et al. (2023)
		Fabrication of green colorimetric smart packaging based on basil seed gum/chitosan/red cabbage anthocyanin for real-time monitoring of fish freshness	Nadi et al. (2023)
		Evaluation of the effect of basil seed gum, tragacanth gum, pectin, and coating formulation with corn flour on oil absorption and sensory properties of watermelon rind chips	Rezagholizade-shirvan et al. (2023)
		Investigating functional properties of halloysite nanotubes and propolis used in reinforced composite film based on soy protein/basil seed gum for food packaging application	Shahabi et al. (2023)
		Investigating the characteristics of basil seed gum-based film enriched with *Echinophora platyloba* extract and its preservative effect on the quality of silver carp	Abdolmaleki et al. (2023)
		Development of active and intelligent colorimetric biopolymer indicator: Anthocyanin-loaded gelatin-basil seed gum films	Shakouri et al. (2023)
		Preparation and physicochemical evaluation of casein/basil seed gum film integrated with guar gum/gelatin based nanogel containing lemon peel essential oil for active food packaging application	Mortazavi Moghadam et al. (2023)
		Atmospheric pressure cold plasma modification of basil seed gum for fabrication of edible film incorporated with nanophytosomes of vitamin D3 and tannic acid	Hashemi Gahruie et al. (2023)
2022	8	Development and characterization of an antimicrobial edible film from basil seed (*Ocimum basilicum* L.) mucilage and sodium alginate	Nazir and Wani (2022)
		Preparation and evaluation some properties of pH indicator film based on Arabic gum- Carboxy methyl cellulose composite film containing of violet basil (*Ocimum basilicum*. L) anthocyanin	Rezaei et al. (2022)
		Different percentages of basil seeds (*Ocimum basilicium* L.) As hydrocolloid in batter coating system: Effect on the physicochemical and sensory properties of breaded fish fillets	Abdul-Zubir et al. (2022)
		Multilayered mucoadhesive hydrogel films based on *Ocimum basilicum* seed mucilage/thiolated alginate/dopamine-modified hyaluronic acid and PDA coating for sublingual administration of nystatin	Sadat Hosseini et al. (2022)
		Effect of basil and chitosan coating on drying kinetic, color, texture and antioxidant activity of apple slices: Hot air oven and vacuum drying	Firouzi et al. (2022)
		Nanoemulsion-based basil seed gum edible film containing resveratrol and clove essential oil: In vitro antioxidant properties and its effect on oxidative stability and sensory characteristic of camel meat during refrigeration storage	Ansarian et al. (2022b)
		Effect of edible coating of Basil seed mucilage with different levels of black Caraway extract on quality and shelf life of lactic cheese	Khodai Kolaleh et al. (2022)
		Coating of zucchini slices with Balangu, basil, and wild sage seeds gums to improve the frying properties	F. Salehi et al. (2022)
2021	9	The antibacterial activity of nano-encapsulated basil and cinnamon essential oils against certain multidrug-resistant bacteria recovered from infected wounds	El-Kattan and Allam (2021)
		Investigation of the chemical properties of *Mentha pulegium* essential oil and its application in *Ocimum basilicum* seed mucilage edible coating for extending the quality and shelf life of veal stored in refrigerator (4 °C)	Tanavar et al. (2021)
		Effect of basil seed and xanthan gums coating on color and surface change kinetics of peach slices during infrared drying	F. Salehi and Satorabi (2021a)
		Basil seed gum edible films incorporated with *Artemisia sieberi* and *Achillea santolina* essential oils: Physical, antibacterial, and antioxidant properties	Hashemi and Khodaei (2021)
		Infrared drying kinetics of coated peach slices with basil seed and xanthan gums	F. Salehi and Satorabi (2021c)
		Effect of hydrocolloid coatings (Basil seed gum, xanthan, and methyl cellulose) on the mass transfer kinetics and quality of fried potato strips	Zamani-Ghalehshahi and Farzaneh (2021)
		Phenolic compounds and antioxidant activities of lemon wastes affected by microencapsulation using coatings of Arabic, Persian, and basil seed gums	Shaygannia et al. (2021)
		Comparing the effect of Arabic, basil seed and *Salvia macrosiphon* gums-based coatings on the shelf-life of tomatoes	Fallah et al. (2021)
		Influence of infrared drying on drying kinetics of apple slices coated with basil seed and xanthan gums	F. Salehi and Satorabi (2021b)
2020	10	Fabrication and characterization of an active bionanocomposite film based on basil seed mucilage and ZnO nanoparticles	Foghara et al. (2020)
		Antimicrobial and antioxidant coating based on basil seed gum incorporated with Shirazi thyme and summer savory essential oils emulsions for shelf-life extension of refrigerated chicken fillets	Majdinasab et al. (2020)
		The effects of fatty acids chain length on the techno-functional properties of basil seed gum-based edible films	Hashemi Gahruie et al. (2020)
		Effect of gamma irradiation on physico-mechanical and structural properties of basil seed mucilage-chitosan films containing Ziziphora clinopodioides essential oil and MgO nanoparticles for rainbow trout packaging	Naeeji et al. (2020a)
		The Influence of Natural Basil Seed Gum Coats on the Kinetics of Osmotic Dehydration of Apple Rings	Etemadi et al. (2020)
		*Ocimum basilicum* seed mucilage reinforced with montmorillonite for preparation of bionanocomposite film for food packaging applications	Rohini et al. (2020)
		In vitro antimicrobial effect of basil seed mucilage-chitosan films containing ziziphora clinopodioides essential oil and MgO nanoparticles	Naeeji et al. (2020b)
		Effect of lemon basil (*Ocimum basilicum*) seed mucilage and Chinese quince (*Pseudocydonia sinensis*) seed mucilage coating to cherry tomato (*Solanum lycopersicum* ‘Sida’) fruits	Inthamat et al. (2020)
		Enrichment of *Aloe vera* gel with basil seed mucilage preserve bioactive compounds and postharvest quality of apricot fruits	Nourozi and Sayyari (2020)
		Property improvement of antibacterial wound dressing from basil seed (*O. basilicum* L.) mucilage- ZnO nanocomposite by borax crosslinking	Tantiwatcharothai and Prachayawarakorn (2020)
2019	5	Effect of basil seed gum based edible coating enriched with echinacea extract on the postharvest shelf life of fresh strawberries	Moradi et al. (2019)
		Study on hydrophobic modification of basil seed gum-based (BSG) films by octenyl succinate anhydride	Hashemi Gahruie et al. (2019a)
		Development, modification and characterization of new biodegradable film from basil seed (*Ocimum basilicum* L.) mucilage	Thessrimuang and Prachayawarakorn (2019)
		Characterization of an antibacterial wound dressing from basil seed (*Ocimum basilicum* L.) mucilage-ZnO nanocomposite	Tantiwatcharothai and Prachayawarakorn (2019)
		Separated and combined effects of nano coating of basil seed gum and perfoliatumseed gum containing kiwi peel extract to increase shelf life of sheep's meat	Pourshaygan et al. (2019)
2017	5	Characterization of novel basil-seed gum active edible films and coatings containing oregano essential oil	Hashemi and Mousavi Khaneghah (2017)
		Characterization of basil seed gum-based edible films incorporated with *Zataria multiflora* essential oil nanoemulsion	Hashemi Gahruie et al. (2017)
		Application of active edible coatings made from basil seed gum and thymol for quality maintenance of shrimp during cold storage	Khazaei et al. (2017)
		Basil-seed gum containing *Origanum vulgare* subsp. viride essential oil as edible coating for fresh cut apricots	Hashemi et al. (2017)
		Basil seed mucilage as a new source for electrospinning: Production and physicochemical characterization	Kurd et al. (2017)
2016	2	Effect of active edible coatings made by basil seed gum and thymol on oil uptake and oxidation in shrimp during deep-fat frying	Khazaei et al. (2016)
		Functionality of coatings with Salep and basil seed gum for deep fried potato strips	Karimi and Kenari (2016)
2015	1	The influence of different plasticisers and fatty acids on functional properties of basil seed gum edible film	Mohammad Amini et al. (2015)
2014	1	Characterization of new biodegradable edible film made from basil seed (*Ocimum basilicum* L.) gum	Khazaei et al. (2014)

The primary objective of this review is to provide a comprehensive overview of the current state of research on basil seed gum-based films and coatings, with a particular emphasis on their role in active packaging and coating systems for food preservation. This review primarily focuses on literature published between 2017 and early 2025, with specific emphasis on research from the last five years (2020–2025), to ensure that the trends and developments presented are up to date and relevant. This includes an in-depth analysis of the physicochemical and mechanical properties and the techniques employed to produce functional films and coatings. The review will delve into basil seed gum-based active packaging development, incorporating bioactive compounds such as antioxidants, antimicrobials, and natural extracts to enhance food quality and extend shelf life. Additionally, the applications of basil seed gum-based coatings in preserving a wide range of food products, including seafoods, fruits, vegetables, meat, poultry, and fried potatoes, will be explored. These materials act as protective barriers, regulating moisture, oxygen, and microbial activity, thereby maintaining the freshness and safety of food products.

Furthermore, the advantages and limitations of basil seed gum as a material for active packaging and coatings will be critically examined. While basil seed gum offers excellent film-forming ability, biodegradability, and compatibility with active agents, water sensitivity and mechanical strength must be addressed. Recent innovations and modifications, such as blending with other biopolymers, cross-linking, or incorporating nanoparticles, will be discussed as strategies to overcome these limitations. By comparing basil seed gum with other polysaccharide-based films, this review aims to highlight its unique properties, such as its superior barrier performance and ability to carry active compounds, which make it a promising candidate for commercialization in the food industry. Through this review, we aim to provide valuable insights into the potential of basil seed gum-based active packaging and coatings to revolutionize food preservation, reduce food waste, and contribute to sustainable packaging solutions. The findings will serve as a foundation for future research and development in this field, addressing scientific and industrial challenges.

## 2 Basil seed gum films

Like other gums, the use of basil seed gum as a packaging material has been widely studied. However, to our knowledge, establishing a comprehensive range of properties for biopolymer-based films and coatings remains challenging. Additionally, direct comparisons between basil seed gum-based films and other biodegradable materials may not always be feasible. However, delving into the native structure and functionality of basil seed gum-based films and coatings may help scientists to have a comprehensive perspective on the potential of this gum-based film as an active packaging material and coating.

The film-forming ability of basil seed gum-based films and their dependence on plasticizers are important considerations. It is worth noting that incorporating basil seed gum with plasticizers is essential to enhance the mechanical and physical properties of the films (Khazaei et al., 2014; Mohammad Amini et al., 2015; Naji-Tabasi & Razavi, 2017). Mohammad Amini et al. (2015) examined the effect of different plasticizers on basil seed gum-based films. Sorbitol endowed a higher hydrophilicity to the film matrix, while a higher moisture content, water vapor permeability, and moisture uptake were observed. However, the application of sorbitol as a plasticizer endowed the films with a lower level of the properties mentioned above. On the other hand, applying fatty acids as plasticizers increased the hydrophobicity of films, demonstrated by a lower moisture uptake, solubility, water vapor permeability, and higher contact angles. In this study, oleic acid successfully enhanced basil seed gum-based films' permeability and mechanical properties compared to saturated fatty acids, such as palmitic and stearic acid. The incorporation of fatty acids with different chain lengths as plasticizers was also investigated by Hashemi Gahruie et al. (2020). They reported a higher chain length resulting in higher thickness and opacity, and lower solubility and water vapor permeability. While the higher chain length resulted in better mechanical and barrier properties, lauric acid could be more effective than caprylic acid. This conclusion might be due to the higher uniformity and permeability of basil seed gum-based films incorporated with lauric acid. Incorporating caprylic and lauric acids into basil seed gum films enhanced their tensile strength due to their even distribution within the polymer matrix. In contrast, palmitic acid showed no such improvement because its high melting point and strong hydrophobicity limited dispersion. None of the fatty acids notably affected elongation at break, indicating they did not act as plasticizers. Despite these results, glycerol remains a widely recognized plasticizer for preparing basil seed gum-based films (Hashemi & Mousavi Khaneghah, 2017; Khazaei et al., 2014; Naji-Tabasi & Razavi, 2017). However, the optimal level of incorporation still requires further investigation. Generally, the incorporation of glycerol successfully enhances the permeability and solubility of the film, as Khazaei et al. (2017) reported. Among the various concentrations of glycerol tested (25 %, 35 %, and 50 % *w*/w of basil seed gum), the highest extensibility was observed with 50 % glycerol incorporation into the film matrix. However, this came at the expense of a significant reduction in tensile strength. The results suggest that incorporating 25 % glycerol could effectively enhance permeability properties and mechanical strength while maintaining uniformity in the microstructure.

### 2.1 Modified basil seed gum films

Edible films in the food industry refer to thin, flexible layers made from edible materials applied directly onto food surfaces. Composed of biopolymers like proteins, polysaccharides, or lipids, these films serve as protective barriers, enhancing food preservation and quality (Bahramian et al., 2025; Mohamed et al., 2020; Shahabi et al., 2023). Their application extends to various purposes, including extending shelf life, preventing moisture loss, and controlling the release of additives. Edible films are considered an eco-friendly alternative to conventional packaging, reducing environmental impact. Widely researched and implemented, they offer innovative solutions for sustainable food packaging, ensuring functional and edible attributes meet evolving consumer preferences and environmental concerns (Asiri et al., 2024). Hydrocolloids and gums are often modified to enhance their functionality and suitability for edible film preparation in the food industry. Modifications are used to tailor specific properties such as film-forming ability, mechanical strength, flexibility, water vapor barrier, and solubility (Hashemi Gahruie et al., 2019b; Sahraeian, Rashidinejad, & Niakousari, 2023). These adjustments may involve chemical, physical, or enzymatic modifications to improve the performance of the hydrocolloids and gums in film applications. For instance, modifications can optimize the film's stability, thickness, and adhesion properties, ensuring it meets the desired food packaging or preservation requirements. Additionally, modified hydrocolloids and gums can exhibit improved resistance to environmental factors, extending the shelf life and overall effectiveness of the edible films in food applications. For example, Salehi, Ghazvineh, and Amiri (2024) and Salehi, Hashemi, et al. (2024) found that modifying basil seed gum with octenyl succinate anhydride improved its compatibility with nanoencapsulated vitamin E compared to native basil seed gum films. This modification led to enhanced mechanical properties, permeability, and antioxidant activity. In detail, incorporating vitamin E into modified basil seed gum films reduced tensile strength, with the lowest values observed for the free form, while encapsulated forms showed no significant difference from each other. All forms enhanced film flexibility, but liposome and especially nanoliposome encapsulation produced the greatest increase in elongation at break, likely due to a lubricating effect that eased molecular movement within the film matrix.

Various approaches have been employed to prepare modified gums with desired functional properties. These modification methods include carboxymethylation, grafting with other biopolymers, cyan ethylation with acrylonitrile, crosslinking, irradiation, and physical processes. Among their numerous applications, preparing modified gum as a packaging material with desired characteristics has also greatly interested researchers (Hosseinzadeh et al., 2020; J. H. Lee et al., 2019; Sahraeian et al., 2024; Zhu et al., 2022).

Moreover, adding biopolymers and crosslinkers, such as proteins or ferric chloride, into the film-forming formulation can be a practical approach for preparing composite films with modified properties. For instance, waxes and fatty acids have been incorporated as hydrophobic ingredients, imparting excellent water vapor permeability (WVP) attributes. However, they can also increase the brittleness and opacity of the film sheets (Chen et al., 2022; Nurul Syahida et al., 2020).

Due to their low miscibility, forming film-forming emulsions with physical stability before drying can be challenging. A strategy to address this problem and enhance the bio-based attributes of the films is through crosslinking, which involves the application of various crosslinkers or irradiation (Hua et al., 2021; Zhou et al., 2021). Notably, some crosslinkers may exhibit toxicity to some extent (Wen et al., 2021).

As mentioned earlier, modifying gums imparts various packaging and coating material characteristics. For example, chemical modification can reduce the hydrophilic nature of gums without marked modification in the bulk structure. Hydrophobic molecules esterify hydroxyl groups and the biopolymer structure to create a hydrophobically modified film network with minimal sensitivity to water (Ali et al., 2017; Kanwar et al., 2021; Park et al., 2021). The characteristics of the modified basil seed gum film are summarized in Table 2.

**Table 2:** Effects of different modification techniques on the functional properties of basil seed gum-based films and coatings. Upward arrows (↑) and downward arrows (↓) indicate an increase and a decrease in the parameters, respectively.

Film formulation	Active components	Main advantages	Probable mechanism or explanation	Reference
Basil seed gum	Oregano essential oil	WVP ↓, Moisture content ↑, contact angle ↑, transparency ↑, and swelling index↑, antibacterial and antioxidant activity ↑	• Reduced bonding ability to water • Enhancement of tortuosity factor of the vapor diffusion-path	(Hashemi et al., 2017)
Basil seed gum modified with tartaric acid (TA), malic acid (MA) and succinic acid (SA)		Swelling index, WVP of crosslinked films > MA, TA, SA Crosslinking: ↑ Strain at maximum load, surface uniformity ↑, Thermal stability ↑ Maximum stress load of crosslinked films ∼ commercial low-density polyethylene (LDPE) film	• Crosslinking effect	(Thessrimuang & Prachayawarakorn, 2019)
Basil seed gum modified with octenyl succinate anhydride		Modified basil seed gum films: Contact angle ↑, density ↑, ultimate strength ↑, opacity ↑, flexibility ↑, solubility in water ↓, and WVP ↓, no significant effect on thickness, moisture content, and color properties	• Presence of hydrophobic octenyl succinate groups	(Hashemi Gahruie et al., 2019b)
Basil seed gum	Zataria multiflora essential oil	Mechanical properties ↑, surface uniformity ↑, antimicrobial properties ↑, and release of volatile compounds ↓	• Formation of more compact structures in the presence of nanodroplets	(Hashemi Gahruie et al., 2017)
Basil seed gum modified with polyvinyl alcohol (PVA) and glutaraldehyde (GA)	Tetracycline hydrochloride	Modified samples: swelling index ↑, water retention capability ↑, and no cytotoxicity. Loading capacity was improved at pH = 8.5 and 7.4 compared to acidic conditions.	• Increased cross-linking degree	(Hosseini & Nabid, 2020)
Basil seed gum modified with caprylic, lauric, and palmitic) acids		WVP ↓, mechanical properties ↓, and color properties ↓ Caprylic acid-modified samples: Surface density ↑ and uniformity ↑ Lauric acid- and palmitic acid-modified samples: Roughness ↑ and insoluble particles ↑ Lauric acid-modified samples were the most acceptable samples.	• The hydrophobic nature of fatty acids resulted in reduced WVP	(Hashemi Gahruie et al., 2020)
Basil seed gum	Zinc oxide nanoparticles	Mechanical properties ↑, thermal properties ↑, water retention capability ↑, and antimicrobial properties ↑, no cytotoxicity, and no adherence	• Nano particles acted as good reinforcing elements	(Tantiwatcharothai & Prachayawarakorn, 2019)
basil seed gum -chitosan	*Ziziphora clinopodioides* essential oil and MgO nanoparticles	Tensile strength ↑, swelling capacity ↓, and WVP ↓.	• Strong chemical reactions between film constituents	(Naeeji et al., 2020a)

**Fig. 2:**
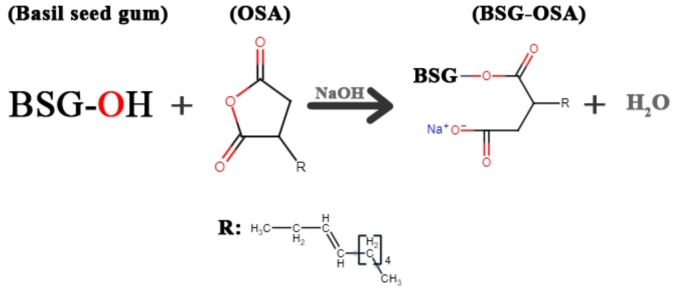
The schematic of the esterification reaction between OSA and basil seed gum.

In a study, Hashemi Gahruie et al. (2019a) examined the hydrophobic modification of basil seed gum-based films using octenyl succinate anhydride. Adding octenyl succinate groups onto the basil seed gum polymer chains was also confirmed through NMR and FTIR analysis. The schematic representation of the esterification reaction between OSA and basil seed gum is shown in Fig. 2. Findings from X-ray diffraction (XRD) analysis data showed no significant physical alterations. Compared with the control film, the hydrophobicity or contact angle of the modified basil seed gum film was higher. Additionally, a reduction in WVP by 50 % and film solubility in water by 29 % was observed. However, opacity increased by 21.37 %, and density increased by 14.28 % after modification at a concentration of 0.03. Fig. 2 shows the schematic representation of the OSA and basil seed gum esterification reaction. Furthermore, the findings revealed that OSA-modified films had no significant impact on the modified films' thickness, color characteristics, and moisture content. There was a substantial increase in the ultimate strength and flexibility of the basil seed gum films modified with OSA at a weight ratio of 0.03. Their findings showed that modifying basil seed gum with OSA is a suitable alternative for developing edible films and coatings with relatively high water resistance.

In another study (2019), specific dicarboxylic acids, such as malic acid, succinic acid, and tartaric acid, were used as crosslinking agents to modify basil seed gum; this modification aimed to alter the chemical structures and acidity and increase the WVP and mechanical properties. FTIR analyzed the crosslinked films, and a 1730 cm^−1^C=O stretching peak was observed, confirming the presence of ester bonds between the polysaccharide backbones and crosslinkers. Crosslinked film samples exhibited a more significant gel fraction and improved WVP than intact samples. Additionally, a better swelling capacity was observed in succinic acid-treated samples than in tartaric acid and malic acid-treated ones. Crosslinked films displayed a more uniform topology, higher thermal stability, and a remarkable increase in the strain at the highest load. Moreover, the maximum stress load was comparable to that of commercial low-density polyethylene film. Overall, the instrumental attributes of basil seed gum films, including outstanding stress and strain at the maximum load, thermal stability, and enhanced barrier properties, were improved through crosslinking with dicarboxylic acids.

Hosseini and Nabid (2020) conducted a study on preparing pH-responsive hydrogel films based on basil seed gum as a new drug delivery system for wound dressing applications. Different formulations of glutaraldehyde and polyvinyl alcohol, as crosslinking agents and a plasticizer (glycerol), were incorporated to achieve a desirable balance of hardness and softness. Examining hydrogel films involved TGA, FTIR, and morphological analysis using SEM. The physical attributes were determined through rheology, mechanical testing (stress at maximum load and Young's modulus), gel fraction, WVP, oxygen permeability, water retention capacity, and swelling capacity measurements. The most favorable results were obtained with the Mu-Gly2 treatment (1 % basil seed gum, 0.5 % glutaraldehyde, 2 % PVA, and 30 % glycerol), which exhibited a suitable gel fraction and swelling degree. This formulation was found to be biocompatible and non-toxic based on the results of cytotoxicity testing. A model drug (Tetracycline hydrochloride) was used to load the optimized film formulations. The release pattern demonstrated more desirable results at pH values of 8.5 and 7.4 compared to acidic pH.

In another investigation by Naeeji, Shahbazi and Shavisi, 2020a, Naeeji, Shahbazi and Shavisi, 2020b, the role of various gamma irradiation levels (2.5 and 5 kGy) in the structural and physico-mechanical attributes of films made from basil seed gum-chitosan was evaluated. Different concentrations of *Ziziphora clinopodioides* (1 % and 2 %) and magnesium oxide nanoparticles (0.1 % and 0.2 %) were incorporated. Compared to the other prepared films, better WVP rates, tensile performance, and lower swelling index were observed when films were exposed to a radiation dose of 5 kGy from a cobalt-60 source. Edible chitosan films with antimicrobial activity were effectively prepared using magnesium oxide nanoparticles (0.1 % and 0.2 %) and *Ziziphora clinopodioides* EOs (1 and 2 %). The results indicated that irradiation improved physicochemical attributes, including WVP, solubility index, tensile strength, and thickness, especially under a 5 kGy dose, compared to the rest of the prepared films. Gamma irradiation at moderate doses (2.5–5 kGy) slightly improved the tensile strength and reduced swelling and WVTR of basil seed mucilage–chitosan films, likely by promoting cross-linking and densifying the film matrix without causing significant polymer degradation.

### 2.2 Basil seed gum-based active films and coatings

#### 2.2.1 Overview

Unlike conventional packaging materials, active packaging interacts with food or its surrounding environment to provide additional functionalities, such as antimicrobial (Khan et al., 2024; Saboktakin-Rizi et al., 2025), antioxidant (Sahraeian et al., 2024), and oxygen-scavenging (Luo et al., 2025) properties. Due to its excellent film-forming ability and compatibility with bioactive compounds, Basil seed gum is an ideal biopolymer for developing active packaging materials (Salehi, Hashemi, et al., 2024). Incorporating biologically active ingredients into the basil seed gum film matrix enhances its functionality, making it a valuable tool for improving food preservation and safety. Integrating bioactive compounds into basil seed gum-based films and coatings enables multifunctional properties, including antimicrobial and antioxidant activities, moisture regulation, and UV protection. The most commonly used bioactive ingredients include:

1. Antimicrobial agents: Basil seed gum-based active films can effectively inhibit microbial growth by incorporating natural antimicrobial compounds, such as essential oils derived from thyme (Çoruhlu et al., 2025; Majdinasab et al., 2020), oregano (Hashemi & Mousavi Khaneghah, 2017), and clove (Ansarian et al., 2022a). These compounds disrupt microbial cell membranes, reducing spoilage and improving food safety.
2. Antioxidant compounds: Oxidation is a major cause of quality deterioration in food products, leading to rancidity and nutrient loss. Antioxidant compounds such as polyphenols (Shakouri et al., 2023), vitamins (Salehi, Ghazvineh, & Amiri, 2024; Salehi, Hashemi, et al., 2024), and carotenoids (Komijani et al., 2022) Basil seed gum films can be incorporated to prevent lipid oxidation and preserve food quality.
3. UV protection and light barrier functionality: Many bioactive compounds, such as anthocyanins (Shakouri et al., 2023) and flavonoids (Al-Musawi et al., 2023), exhibit UV-absorbing properties. Incorporating these agents into basil seed gum-based films can protect food components from light-induced degradation.
4. Natural dyes: Using pH-sensitive natural dyes such as anthocyanins in basil seed gum films allows real-time monitoring of food freshness (Kafashan & Babaei, 2024). Colorimetric changes in the film can indicate spoilage, providing a visual cue for consumers.

The following sections discuss in detail the application of bioactive compounds in basil seed gum-based films and coatings.

### 2.3 Incorporation of bioactives

#### 2.3.1 Basil seed gum films

Essential oils are intriguing candidates for addition to basil seed gum-based composite films due to their notable antimicrobial and antioxidant activities. Essential oils are commonly used as flavoring agents in food formulations. Many studies have reported that essential oils exhibit antimicrobial activities against food spoilage and pathogenic microorganisms (Alqahtani et al., 2024; Chouhan et al., 2017; Ilić et al., 2022; Wang, Wang, et al., 2023; Wang, Yu, et al., 2023). However, it is essential to consider various factors when incorporating essential oils into food systems, primarily due to flavor considerations and certain limitations, including high volatility, hydrophobicity, and potential interactions with other ingredients (Falleh et al., 2020). In more detail, essential oils need to be emulsified in the formulation to create antimicrobial films. Therefore, the particle size of emulsified essential oils is expected to affect their functionalities significantly. Nano-sized emulsions less than 100 nm possess two remarkable features: improved stability and enhanced physicochemical activity (Jamali et al., 2021). Secondly, biological activities are enhanced by increasing the specific surface area, reducing the required amount of bioactive (Li, Jiang, et al., 2022). Numerous studies have reported significant advancements in the antibacterial and antioxidant activities of nanoemulsions loaded with essential oils compared to conventional emulsions (Jamali et al., 2021; Li, Jiang, et al., 2022; Sahraeian et al., 2024; Sharma et al., 2022; Zhang et al., 2024).

Therefore, a common method to reduce WVP in composite films is to mix biopolymer-based solutions with hydrophobic components such as waxes, fats, oils, fatty acids, and fatty alcohols. A study by Hashemi Gahruie et al. (2017) investigated the incorporation of *Zataria multiflora* essential oil (*ZM*EO) nanoemulsion into basil seed gum edible films. Their objective was to enhance the bioactivity of *ZM*EO by producing a nanoemulsion and immobilizing it within the basil seed gum-based film network. Different sonication times (0, 2.5, 5, and 10 min) at high intensity (150 W) were employed to prepare *ZM*EO nanoemulsions. As the antimicrobial activities of the *ZM*EO nanoemulsion increased, the nanoemulsion droplet size decreased. Increased nanoemulsion content of basil seed gum films led to improved mechanical stability. Scanning electron microscopy results indicated that incorporating *ZM*EO nanoemulsions could significantly alter the basil seed gum films' topography. The fabricated antimicrobial films were found to be effective against a variety of foodborne pathogens. Incorporating Eos into biopolymer-based films is considered a novel approach that can extend the shelf life of food products by enabling the slow release of volatile components.

Similarly, Hashemi and Mousavi Khaneghah (2017) investigated the incorporation of oregano EO into basil seed gum-based edible coating films. They aimed to create films with antibacterial activity against *Salmonella Typhimurium*, *Escherichia coli*, *Pseudomonas aeruginosa*, *Bacillus cereus*, and *Staphylococcus aureus*. They evaluated various properties of the films, including swelling capacity, antioxidant properties, film thickness, transparency, contact angle, and WVP. Incorporating oregano, EO significantly reduced WVP while swelling index, moisture content, transparency, and contact angle increased. All treatments exhibited significant antimicrobial and antioxidant activities. The results indicated that this edible coating can be applied to food packaging. Incorporating bioactive substances into basil seed gum films has not been limited to Eos. For example, Hashemi Gahruie et al. (2020) investigated whether the fatty acid chain length affects the functional characteristics of basil seed gum films. They studied the effects of fatty acids, including caprylic, lauric, and palmitic acids, on basil seed gum-based edible films' physicochemical, barrier, and mechanical stability. FTIR confirmed the esterification of fatty acids in the basil seed gum-based matrix. As the chain length of the esterified fatty acids increased, the films became thicker and opaque. However, water solubility and WVP significantly decreased. When caprylic acid and lauric acid were incorporated, the edible films exhibited improvements in color and mechanical properties, as well as a favorable homogeneous microstructure and higher surface density compared to those incorporated with lauric acid and palmitic acid, which showed excessive roughness and insoluble elements according to the scanning electron microscopy micrographs. So, lauric acid was a suitable alternative for enhancing basil seed gum film's textural and barrier attributes. In addition to the technological applications of basil seed gum-based enriched films in the food industry, their potential medical applications have also been investigated.

For instance, using the lyophilization technique, Tantiwatcharothai and Prachayawarakorn (2019) studied basil seed gum as an antimicrobial wound dressing. They introduced varying amounts of zinc oxide nanoparticles (ZnO-NP) as an antimicrobial agent. Basil seed gum hydrogel exhibited excellent porosity and swelling capacity. The results of FTIR analysis revealed interactions among basil seed gum molecules and ZnO-NP. Scanning electron microscopy data indicated an integrated open-pore structure in basil seed gum hydrogels with well-distributed ZnO-NP. Furthermore, increased ZnO-NP content improved mechanical attributes (strain at maximum load of 51 %, Young's modulus of 151 MPa, and stress at maximum load of 8.9 MPa), water retention capacity, thermal stability, and antibacterial activity. Cytotoxicity results and cell adhesion tests showed that basil seed gum hydrogel was non-adherent and non-cytotoxic.

Basil seed gum-based enriched composite films have demonstrated promise as a substrate for developing practical materials with a wide range of potential applications. To enhance the quality of these films and expand their applications, further research is required to optimize their formulation and processing conditions.

#### 2.3.2 Basil seed gum coatings

In the food industry, coating refers to a thin layer applied to the surface of a food product for various purposes, such as enhancing appearance or providing a protective barrier. Coatings can be made from multiple materials, including edible substances like hydrocolloids (Eftekhari et al., 2023; Pavlath & Orts, 2009; Wang, Wang, et al., 2023; Wang, Yu, et al., 2023). The key distinction between a film and a coating lies in their application and composition. While both are thin layers, films are typically standalone layers that completely envelop a food item, often as a barrier or wrapper. On the other hand, coatings are thinner and may only partially cover the food, usually applied for specific purposes like glazing or sealing.

**Fig. 3:**
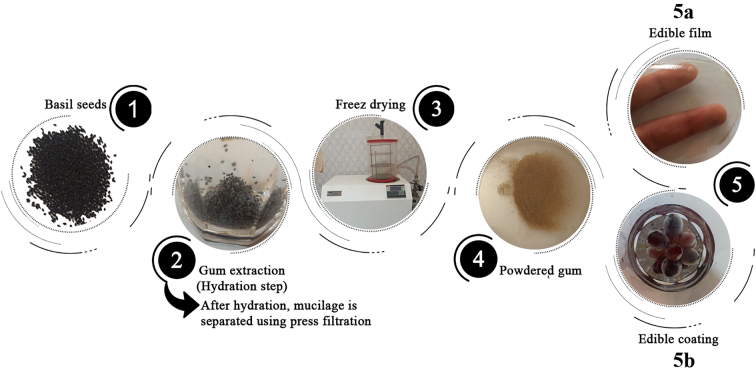
Preparation of basil seed gum for edible films and coatings.

Food edible coatings made of gum have drawn much attention recently because of their potential to maintain food quality, extend shelf-life, and improve the safety of food products. These coatings are made of natural gums such as basil seed gum, which act as a barrier to shield the food surface from oxidation, microbial growth, and moisture loss. Basil seed gum-based coatings can be applied using various techniques, including dipping, spraying, or brushing, to different food products, including fruits, vegetables, meats, and seafood. Depending on the type of food product and its intended usage, the characteristics of the coating, such as thickness, composition, and adherence, can be modified for specific needs. Moreover, coating materials with additional properties can be a suitable alternative to synthetic preservatives. For example, the application of antimicrobial coatings has received much attention. It is widely studied, mainly due to its ability to provide improved protection for sensitive food products during cold storage (Chawla et al., 2021; Hernández-García et al., 2021; Priyadarshi et al., 2022; Sahraeian, Niakousari, et al., 2023). To prepare the basil seed gum, seeds are impregnated in water at a mild temperature and allowed to swell, surrounded by a mucilage layer. The mucilage is then separated using filtration, followed by freeze-drying or oven-drying (Fig. 3). The following sections discuss the application of basil seed gum-based coatings for various food products.

## 3 Application for food preservation

### 3.1 Seafood coating

In recent years, seafood quality and safety have become growing concerns in the food industry. Customers increasingly prefer food products with minimal levels of synthetic preservatives, microbes, and oxidants (Baptista et al., 2020; Saldanha Pinheiro et al., 2023). Direct application methods such as spraying or dipping have been employed for shrimp meat (Dayakar et al., 2022; Gokoglu et al., 2022). However, due to several limitations, preservatives applied directly to the surface can neutralize bioactive compounds, evaporate, or diffuse insufficiently into foods (Mousavi Khaneghah et al., 2018). Active coatings containing bioactive compounds, including antimicrobials, antioxidants, vitamins, colorants, and flavors, have been developed to enhance food appearance and extend its shelf life (Omerović et al., 2021). The coating method creates a more stable antimicrobial environment, allowing for a controlled release of the antimicrobial agents and leading to sufficient microbial inhibition. In contrast, a burst release can temporarily inhibit (Deshmukh & Gaikwad, 2024; He et al., 2025).

Seafood coatings can be made from various ingredients, such as starch, proteins, and gums (Rathod et al., 2021; Sayyari et al., 2021). Using gums, especially basil seed gum-based coatings, has been shown to enhance the sensory properties of seafood products, including crispiness, crunchiness, and juiciness, while extending their shelf life and reducing waste. Additionally, coatings can deliver bioactive compounds, such as omega-3 fatty acids, to the seafood surface, resulting in improved nutritional quality (Kanelaki et al., 2022; Sun et al., 2022). The effects of fennel EO incorporated into different types of basil seed gum coatings, including nanoemulsion, on extending the shelf life of fish meat were studied by Sayyari et al. (2021). They found that the antioxidant activity of fennel EO increased with increasing concentration, and the basil seed gum nanocoatings improved the antimicrobial properties of the coating. The best results were obtained using a nanocoating of basil seed gum with 2 % fennel EO, which could be used in fish fillet packaging units to improve the shelf life of fish meat. Moreover, basil seed gum coating is a potential medium for the nanoemulsion of EOs, exhibiting effective preservative characteristics. Preserving fried seafood presents challenges as it can lead to changes in texture, flavor, and nutritional quality. Furthermore, using preservatives and additives may alter the sensory and health aspects of the seafood, making it less desirable for consumption. Khazaei et al. (2016) examined the effects of basil seed gum active coating treatments containing thymol on the quality of fried shrimp. This study investigated oil absorption, moisture loss, lipid oxidation, and organoleptic evaluation of fried shrimp. The coating treatments reduced oil uptake, moisture loss, and lipid oxidation by up to 46.4 % for peroxide value (PV) and 40.8 % for thiobarbituric acid (TBA). Regarding organoleptic properties, the coated samples exhibited lower toughness and stiffness and higher acceptability for texture, juiciness, chewiness, and overall acceptability. There was no discernible color, smell, or taste difference across treatments. In another study, basil seed gum active coatings were developed to enhance the quality of *Litopenaeus vannamei*, also known as Pacific white shrimp. Also, the impacts of different coating solutions containing thymol on the quality of the shrimp during two weeks of refrigeration were evaluated. The active coatings delayed the occurrence of the maximum threshold of TVB-N (300 mg/kg). They significantly reduced microbial growth in stored shrimp without adversely affecting the sensorial properties (Khazaei et al., 2017).

Gum coatings for seafood have been found to provide various health benefits, including reducing oil absorption and lipid oxidation in fried seafood products and improving microbiological quality and sensory characteristics. Gum coatings, such as basil seed gum coatings, can potentially enhance food safety and extend the shelf life of marine products. Further research is needed to refine the formulation and application of gum coatings to ensure their effectiveness in various seafood products.

### 3.2 Fruits and vegetables

In recent years, novel techniques have been developed to preserve fresh-cut fruit. Fruits and vegetables are inherently perishable, especially after harvest, due to their active metabolisms, which naturally result in the loss of energy reserves through respiration and water through transpiration (Olivas & Barbosa-Cánovas, 2009). Additionally, various factors, such as biochemical changes, mechanical damage, diseases, pests, and physiological disorders resulting from temperature variations or inappropriate storage atmospheres, contribute to quality loss. Food technologists face challenging issues that include the development of technologies to improve the sorting of fruits and vegetables and to find approaches to sustaining post-harvest quality. In this regard, edible coatings have emerged as a viable approach, promisingly extending the storage life of fresh horticultural products (Ncama et al., 2018; Öztürk, 2025).

A practical approach to enhance the quality and prolong the shelf life of these products involves using coatings to inhibit oxidation, reduce respiration rates, minimize weight loss, and control microbial proliferation (Czerwiński et al., 2021; Maringgal et al., 2020; F. Salehi, 2020a). It should be noted that achieving these results may not be feasible without applying preservatives (Li, Tang, et al., 2022).

The effectiveness of gum coatings varies depending on the type of fruit and the coating formulation. Common gums used for coatings include chitosan, alginate, and basil seed gum. Basil seed gum coatings containing extracts and EOs have been applied to fresh fruits to improve their shelf life and preserve nutritional quality. These coatings offer additional benefits, such as antimicrobial and antioxidant properties, which can help reduce microbial growth and oxidative damage and maintain freshness. Common extracts and EOs used in these coatings include grape seed extract, oregano oil, and cinnamon oil. For instance, Moradi et al. (2019) examined the shelf life of strawberries using a basil seed gum-based coating containing Echinacea extract at different concentrations (0.5 %, 1.5 %, and 3 %) up to 20 days of storage. Samples formulated with 3 % basil seed gum +3 % EE were found to have the lowest microbial count, phenol and anthocyanin degradation, weight loss, and ascorbic acid loss. The strawberries coated with the *Echinacea* extract coating exhibited the maximum antioxidant and superoxide dismutase activity and the highest sensory ratings while having the lowest peroxidase activity. Compared to uncoated ones, the improvement in antimicrobial activity and extended shelf life of coated strawberries over 20 days was attributed to adding *Echinacea* extract into the coating formulation.

Nourozi and Sayyari (2020) enriched *aloe vera* gels (AVG) with basil seed gum to conserve bioactive components and preserve the postharvest quality of apricot fruits. The effects of enriching coatings with AVG and basil seed gum, alone or in combination, on the qualitative properties of apricot fruit were evaluated during 28 days of storage at 2 °C. Coating the apricot fruits with AVG and basil seed gum, either separately or in combination, significantly reduced weight loss, respiration rate, soluble solid content, ripening index, and ethylene production in apricots. Additionally, the titratable acidity and firmness of the coated fruits were higher than those of the control samples. AVG and basil seed gum significantly enhanced the total phenolic content, antioxidant activity, and ascorbic acid levels in apricot fruits during storage at 2 °C. Notably, AVG and basil seed gum had no adverse effects on organoleptic attributes, such as flavor and appearance. These findings suggest that the combination of basil seed gum and AVG and their separate application can be considered successful methods for maintaining postharvest quality and controlling the physiological processes of apricots during refrigerated storage. One limitation of sucrose in osmotic dehydration is the potential for a high sugar content in the final product, which may not be desirable for some consumers. Moreover, the elevated sugar content can increase the risk of microbial growth, necessitating additional processing steps.

In a study by Etemadi et al. (2020), the effect of basil seed gum concentration as a coating solution during the osmotic dehydration process of apple rings was examined. Various parameters, including water loss, solid gain, water and solid diffusion coefficients, and performance ratio, were studied at different concentrations and temperatures of sucrose solutions. The optimal operating condition for achieving the highest performance ratio was a 0.3 % (wt) gum concentration and 60 % (wt) osmotic sucrose solution at 60 °C. Coated samples exhibited reduced sucrose absorption (18 %) compared to the control sample. This study demonstrated that coating pretreatment significantly enhanced the water loss of apple rings. When the basil seed gum coating layer was applied, not only was a more significant reduction in water content from the apple rings achieved, but low sucrose permeability was also achieved, enhancing the dehydration performance. Gum coatings containing EOs have emerged as a natural and effective alternative for preserving fruits and vegetables. These coatings offer antimicrobial and antioxidant properties, inhibiting microbial growth and reducing oxidative damage during storage. EOs commonly used in these coatings include oregano, cumin, thyme, and cinnamon.

In a study by Hashemi et al. (2017), the chemical attributes, sensory characteristics, and microbial load of basil seed gum-containing oregano (*Origanum vulgare*) EO edible coatings for fresh-cut apricots were investigated during 8 days of storage at 4 °C. The results indicated that adding oregano EO significantly reduced the WVP of films while enhancing their moisture content. Also, the coatings reduced the total microbial count, including yeasts and molds. Compared to the control samples, the EO-added samples' antioxidant activity and total water-soluble phenolic content significantly increased during cold storage. The odor and overall acceptability of the samples noticeably improved, suggesting that this novel approach can effectively maintain the quality of fresh-cut apricots.

The postharvest quality and shelf-life of cherry tomatoes (*Solanum lycopersicum* L.) as a result of applying cumin EO-loaded basil seed gum edible coating were examined in a study by Tabaestani et al. (2013). The edible basil seed gum coating containing cumin EO was applied to enhance cherry tomatoes' storability and postharvest quality. Samples were stored for 9 days at 20 °C with a relative humidity of 80–90 %. At the end of the storage period, cherry tomato samples were randomly selected, and their physicochemical properties were examined. The results indicated that, during the storage period, tensile strength decreased for all samples except those treated with EO. Notably, titratable acidity was a significant drop as a function of storage time for all treatments, especially for the basil seed gum coating samples, which showed the highest effectiveness in reducing weight loss during storage. Additionally, the coating had no significant impact on maintaining firmness. Coated samples exhibited acceptable sensory properties, indicating that they preserved the overall quality of cherry tomato fruits during storage. These findings suggest that applying basil seed gum as an edible coating increases weight loss and the shelf life of cherry tomatoes.

### 3.3 Meat and poultry coating

Prolonging the shelf life of fresh and processed meats has posed significant challenges in the food industry. The use of chemical preservatives such as sodium nitrate, benzoic acid, and potassium sorbate has been shown to have undesirable effects on consumers' health. Consequently, recent investigations have shifted their focus toward applying natural preservatives to enhance the shelf-life of meat products, such as fresh poultry, and delay lipid oxidation (Rathod et al., 2023; Wen et al., 2024).

These natural preservatives include EOs (Takma & Korel, 2019), organic acids (Ben Braïek & Smaoui, 2021), bacteriocins (Bhattacharya et al., 2022), modified atmosphere packaging (Kolbeck et al., 2020), and chitosan (Noori & Hossaeini Marashi, 2023). EOs derived from plants, serving as natural antimicrobial agents, have also been widely studied. In a study by Majdinasab et al. (2020), the effect of adding summer savory and Shirazi thyme EOs to basil seed gum coatings on refrigerated chicken fillets was investigated. The coatings significantly improved the quality of the chicken fillets, with the best results observed in samples coated with basil seed gum coatings incorporated with Shirazi thyme EO. These coatings not only reduced microbial growth but also led to decreases in pH, TVB-N, PV, and TBA values while simultaneously enhancing sensory properties compared to the uncoated control samples. Notably, the coating containing Shirazi thyme EO was more effective than the one containing summer savory EO, and no synergistic effect was observed between the two EOs. The study highlights the potential of naturally active components found in EOs in extending the shelf life of coated chicken fillets.

In a study conducted by Rashidaie Abandansarie et al. (2019), rosemary extracts, whether in free form or encapsulated in basil seed gum coating, were employed to enhance the shelf-life of beef meat during refrigerated storage. The study revealed that rosemary extract, particularly in nano-encapsulated form, exhibited significant antimicrobial and antioxidant properties, effectively delaying microbial spoilage and lipid oxidation in beef meat fillets. The most promising results were observed with nano-encapsulated rosemary extract at a concentration of 1600 ppm, extending the fillets' shelf-life by up to 21 days. Encapsulation of rosemary extract in basil seed gum coating enhanced its effectiveness as a preservative, particularly when the extract was nanoemulsified.

Similarly, in a study by Farahmandfar (2019), the effects of nanocoating using basil seed gum and perfoliatum seed gum containing kiwi peel extract were examined to extend the shelf-life of sheep meat. The kiwi peel extract exhibited high antioxidant and antimicrobial activities, particularly at a concentration of 2000 ppm. Using nanotechnology to reduce the size of the coating resulted in improved antioxidant and antimicrobial properties. The composite nanocoating, comprised of basil seed gum and alyssum seed gum containing kiwi extract at a concentration of 2000 ppm, proved to be effective in reducing the chemical and microbial growth rates in sheep meat during refrigerated storage, suggesting its potential utility in the meat packaging industry.

### 3.4 Fried potato coating

Fried potatoes, which are deep-fried, are widely used to prepare ready-to-eat food products. Deep frying involves heat and mass transfer, resulting in various physical and chemical alterations. Substantial heat transfer contributes to the favorable organoleptic attributes of fried food products (Salehi, 2020b).

During the deep-frying process, water vaporization due to thermal treatment creates pores inside the food, increasing the oil content. Given the association of excessive oil consumption with cardiovascular diseases, diabetes, obesity, cancer, and hypertension, the food industry has a significant demand to develop healthier products with reduced oil content. This challenge has motivated scientists to innovate machinery, food ingredients, and novel coating techniques to decrease the oil content in deep-fried products (Flores et al., 2024; Ganesan & Xu, 2020).

They coated food with gum before deep frying, which has emerged as a popular technique used in research to enhance fried foods' texture, flavor, and appearance. Gum coatings create a uniform and adhesive layer on the food surface, effectively preventing oil penetration during frying, which results in a crispy and less greasy final product. Additionally, gum coatings can extend the shelf life of fried foods by reducing moisture loss and preventing oxidation, making them invaluable tools in producing high-quality fried foods. In addition to the previous study discussed in Section 3.1 regarding shrimp coating before deep-frying, Karimi and Kenari (2016) evaluated the effects of basil seed gum and salep coatings on reducing oil absorption in deep-fried potato strips. The coating formulations exhibited pseudoplastic and shear-thinning characteristics, with the most effective formulation being 0.5 % basil seed gum. Consistent with the previous study, basil seed gum coating reduced oil uptake by up to 28.8 %, increasing moisture content and frying yield. It was also observed that the type of oil significantly influenced oil uptake. This study underscores the potential of using basil seed gum-based coatings to reduce oil absorption in fried foods. As is evident, the primary objective of utilizing basil seed gum coating before frying is to reduce the oil absorption of food during the frying process.

In another study conducted by Zamani-Ghalehshahi and Farzaneh (2021), the effects of various hydrocolloids, including coatings and frying temperatures, on the physicochemical properties of potato strips during deep-fat frying were examined. The properties investigated included oil uptake, moisture loss, color, microscopic structure, activation energy, and texture. The study revealed that coating potato strips with a mixture of basil seed gum and xanthan gum (50:50) or basil seed gum alone resulted in the lowest oil uptake. At the same time, the control samples exhibited the highest oil absorption. Consequently, the study concluded that basil seed gum-coated potato strips hold promise as a product with low oil uptake and similar organoleptic properties to the control sample.

In summary, while limited research is available on applying basil seed gum coating before frying food, the existing studies suggest that basil seed gum coating can effectively reduce oil absorption in fried foods and mitigate the adverse effects of frying on food's physicochemical and organoleptic properties. Further research is warranted to explore the impact of basil seed gum coating on various types of foods and to optimize the coating process for industrial applications. Table 3 presents an overview of basil seed gum coating applications.

**Table 3:** Food product applications of basil seed gum-based films and coatings with bioactive agents. Upward (↑) and downward (↓) arrows indicate an increase and decrease in the parameters, respectively.

Coating formulation	Active component	Product	Main advantages	Reference
Basil seed gum	Thymol	Shrimp	Deep-fried shrimp: Oil uptake ↓, lipid oxidation ↓, moisture loss ↓, toughness, and stiffness ↓. No remarkable change in organoleptic attributes. Overall acceptability ↑, juiciness ↑, chewiness ↑, and texture ↑.	(Khazaei et al., 2016)
Basil seed gum and Salep		Potato	Deep-fried potatoes: Oil uptake ↓, moisture content ↑, and frying yield ↑. Different oils led to different oil uptake.	(Karimi & Kenari, 2016)
Basil seed gum	*Origanum vulgare* essential oil	Apricot	WVP ↓, antimicrobial properties ↑, antioxidant properties ↑, moisture content ↑, and total soluble phenolic ↑. Overall acceptability ↑ and odor ↑.	(Hashemi et al., 2017)
Basil seed gum - *Aloe vera*		Apricot	Respiration rate ↓, ripening index ↓, soluble solid content ↓, and ethylene production ↓. Ascorbic acid ↑, firmness ↑, antioxidant activity ↑, titratable acidity ↑, and total phenolic content ↑. No remarkable changes in the organoleptic properties of the fruit.	(Nourozi & Sayyari, 2020)
Basil seed gum	Echinacea extract	Strawberry	Weight loss ↓, phenol and anthocyanin degradation ↓, softening ↓, microbial count ↓, peroxidase activity ↓, and ascorbic acid ↓. Superoxide dismutase activity ↑, antioxidant properties ↑, and organoleptic acceptability ↑.	(Moradi et al., 2019)
Basil seed gum	Shirazi thyme and summer savory essential oils	Chicken fillet	Antimicrobial properties ↑ and antioxidant activity ↑. TVB-N ↓, TVC ↓, TBA ↓, PV ↓, LAB ↓, pH ↓, PBC ↓. Organoleptic attributes ↑.	(Majdinasab et al., 2020)
Basil seed gum	Thymol	Shrimp	Time of reaching the TVB-N threshold ↓and microbial count ↓. No remarkable change in organoleptic attributes.	(Khazaei et al., 2017)
Basil seed gum and *perfoliatum* seed gum	Kiwi peel extract	Sheep's meat	Microbial count ↓, thiobarbituric acid↓, and peroxide value↓.Antioxidant properties ↑ and antimicrobial activities ↑.	(Farahmandfar, 2019)
Basil seed gum	Cumin essential oil	Cherry tomato	Weight loss ↓. No change in firmness. Remarkably changed color properties. Sustained the quality of cherry tomato	(Tabaestani et al., 2013)
Basil seed gum	Thymol	Shrimp	Oil uptake ↓, oxidation ↓, moisture loss ↓, PV ↓, TBA ↓, toughness↓, and stiffness ↓. Scores of overall acceptability ↑, chewiness↑, juiciness↑, and texture ↑.	(Khazaei et al., 2016)
Basil seed gum		Apple ring	Basil seed gum coating was exploited as a barrier in osmotic dehydration. Process performance ↑. Sucrose absorption ↓	(Etemadi et al., 2020)
basil seed gum -chitosan	*Ziziphora clinopodioides* essential oil and MgO nanoparticles	Rainbow trout	Microbial count ↓, TVB-N ↓, and PV ↓.	(Naeeji et al., 2020a)

## 4 Challenges and future prospective

Although basil seed gum demonstrates considerable potential in food packaging systems, there are yet several challenges for food technologists to utilize it in the food industry. According to the literature, basil seed gum-based films have been studied over past decade, indicating it as a novel source of gum for edible food packaging and coating materials in comparison to the commercially used gum such as agar, alginate, guar, and gum Arabic. This reflects a diverse unexplored aspect of biodegradable films based on basil seed gum. In detail, several factors may contribute to this limited attention. For example, basil seeds are not cultivated on a large industrial scale outside of certain Asian countries, which restricts the availability and economic feasibility of this gum for broader applications (Nadeem et al., 2020).

In several studies, including those referenced in this review, freeze-drying is used during the extraction or preparation of basil seed gum to preserve its structural and functional attributes. However, freeze-drying is highly energy-intensive, time-consuming, and costly, making it unsuitable for commercial-scale film or coating production. Its reliance on low temperatures and vacuum pressure systems increases operational costs significantly. Therefore, its utility is largely restricted to laboratory or pilot-scale research. For industrial applications, alternative drying methods such as oven-drying, convective air-drying, or spray-drying may offer more viable and scalable options. These methods require further optimization to balance cost-efficiency with preservation of the gum's functional properties, particularly its film-forming ability and viscosity.

The hydrophilic nature of basil seed gum can be considered as one of the primary challenges. For example, basil seed gum is reported to have a higher affinity to water molecules in comparison with other commercially used gum such as guar gum, flaxseed gum, and artemisia seed gum (Guan et al., 2024). In biopolymers, the higher hydrophilicity compromises water vapor barrier capacity and mechanical strength of packaging systems. This limitation might be more considerable in the packaging of high-moisture food products or hygroscopic food products where their shelf-life might be reduced in humid storage conditions. The surface hydrophilicity can be alleviated through the reduction of surface energy which might be started by increasing the surface roughness (Cui et al., 2023). Recent techniques have been proposed to reduce the hydrophilicity of biopolymer. These techniques include the addition of hydrophobic substances such as essential oils (Saboktakin-Rizi et al., 2025), film's surface modification through vapor deposition (Mostafavi et al., 2023), self-assembly (Xie et al., 2024), and layer-by-layer deposition (Abbadessa et al., 2023).

From a production perspective, the scalability of the production of basil seed gum-based films has yet remained unexplored. According to the literature, published data is limited to the laboratory scale. This might be due to difficulties in the determination of cost efficiency, machinery compatibility, as well as optimum gum extraction, purification and film-forming formulation on an industrial scale. Therefore, further studies should be conducted to generalize and upscale these factors from laboratory scale to pilot plant scale and then industrial scales are demanding. From a critical point of view, finding an optimum, cost effective, and scalable extraction technique might be a practical hike. Regulatory limitations should not be underestimated, however. While it is acknowledged that basil seed gum is extracted from an edible plant, the use of basil seed gum in food-contact materials may require specific safety evaluations, such as toxicological assessments, to comply with food safety regulations in different jurisdictions.

## 5 Conclusion and future scope

Applying films and coatings based on basil seed gum has demonstrated promising potential in enhancing the quality and extending the shelf life of seafood, meat, poultry, fruits, and fried food products. Using basil seed gum as a natural and biodegradable material offers a sustainable alternative to the synthetic coatings commonly used in the food industry. Films and coatings based on basil seed gum have been found to possess excellent barrier properties against moisture, oxygen, and microbial growth, which can significantly reduce the risk of spoilage and extend the shelf life of food products. Additionally, these films and coatings can improve the texture and appearance of food products, providing a more appealing and desirable product for consumers.

One of the significant advantages of employing basil seed gum in food coatings and films is its natural and safe nature. Derived from plant sources, they are non-toxic and non-allergenic, rendering them suitable for application in food products. Moreover, they have been found to have beneficial health effects, such as reducing cholesterol levels and improving digestion, which can appeal to health-conscious consumers. Using basil seed gum as a film-forming agent and coating benefits the food industry economically. As a natural and renewable resource, basil seed gum is relatively inexpensive compared to synthetic coatings, which can be costly and may have adverse environmental impacts. Furthermore, these natural coatings can reduce food waste and losses, resulting in significant cost savings for producers. In conclusion, films and coatings based on basil seed gum have demonstrated the potential to enhance the quality and extend the shelf life of various food products, including seafood, meat, poultry, fruits, and fried foods. Their natural and safe properties make them suitable for use in food products, and their economic benefits can provide cost savings for food producers. Further research is needed to optimize the formulation and application of these coatings to ensure their effectiveness and potential for widespread adoption in the food industry.

## CRediT authorship contribution statement

**Shahriyar Sahraeian:** Writing – review & editing, Writing – original draft, Resources, Funding acquisition. **Hadi Hashemi:** Writing – review & editing, Writing – original draft, Supervision, Software, Project administration. **Fatemeh Ghiasi:** Writing – original draft, Validation, Resources, Investigation. **Mohammad-Taghi Golmakani:** Writing – review & editing, Visualization, Supervision, Project administration. **Shahriyar Valizadeh:** Writing – original draft, Investigation, Funding acquisition, Formal analysis. **Reza Tahergorabi:** Validation, Resources, Methodology. **Yana Artemovna Firsukova:** Writing – original draft, Validation, Investigation. **Amin Mousavi Khaneghah:** Writing – review & editing, Writing – original draft, Supervision, Project administration, Conceptualization.

## Consent to participate

The authors declare their Consent to Participate in this article.

## Declaration of competing interest

The authors declare that they have no known competing financial interests or personal relationships that could have appeared to influence the work reported in this paper.

## Data Availability

No data was used for the research described in the article.
